# Associations between urinary iodine concentration and the prevalence of metabolic disorders: a cross-sectional study

**DOI:** 10.3389/fendo.2023.1153462

**Published:** 2023-05-08

**Authors:** Xia Shen, Long Yang, Yuan-Yuan Liu, Xue-He Zhang, Ping Cai, Jian-Feng Huang, Lei Jiang

**Affiliations:** ^1^ Department of Nursing, Wuxi Medical College, Jiangnan University, Wuxi, China; ^2^ College of Pediatrics, Xinjiang Medical University, Urumqi, China; ^3^ Department of Cardiology, First Affiliated Hospital of Xinjiang Medical University, Urumqi, China; ^4^ Department of Cardiothoracic Surgery, Affiliated Hospital of Jiangnan University, Wuxi, China; ^5^ Department of Radiology, Affiliated Hospital of Jiangnan University, Wuxi, Jiangsu, China; ^6^ Department of Radiology, The Convalescent Hospital of East China, Wuxi, China

**Keywords:** iodine, metabolism, metabolic disorders, epidemiology, NHANES

## Abstract

**Background:**

Few studies have examined the role of iodine in extrathyroidal function. Recent research has shown an association between iodine and metabolic syndromes (MetS) in Chinese and Korean populations, but the link in the American participants remains unknown.

**Purpose:**

This study aimed to examine the relationship between iodine status and metabolic disorders, including components associated with metabolic syndrome, hypertension, hyperglycemia, central obesity, triglyceride abnormalities, and low HDL.

**Methods:**

The study included 11,545 adults aged ≥ 18 years from the US National Health and Nutrition Examination Survey (2007–2018). Participants were divided into four groups based on their iodine nutritional status(ug/L), as recommended by the World Health Organization: low UIC, < 100; normal UIC, 100-299; high UIC, 300-399; and very high, ≥ 400. The Odds ratio (OR) for MetS basing the UIC group was estimated using logistic regression models for our overall population and subgroups.

**Results:**

Iodine status was positively associated with the prevalence of MetS in US adults. The risk of MetS was significantly higher in those with high UIC than in those with normal UIC [OR: 1.25; 95% confidence intervals (CI),1.016-1.539; *p* = 0.035). The risk of MetS was lower in the low UIC group (OR,0.82; 95% CI: 0.708-0.946; *p* = 0.007). There was a significant nonlinear trend between UIC and the risk of MetS, diabetes, and obesity in overall participants. Participants with high UIC had significantly increased TG elevation (OR, 1.24; 95% CI: 1.002-1.533; *P* = 0.048) and participants with very high UIC had significantly decreased risk of diabetes (OR, 0.83; 95% CI: 0.731-0.945, *p* = 0.005). Moreover, subgroup analysis revealed an interaction between UIC and MetS in participants aged < 60 years and ≥ 60 years, and no association between UIC and MetS in older participants aged ≥ 60 years.

**Conclusion:**

Our study validated the relationship between UIC and MetS and their components in US adults. This association may provide further dietary control strategies for the management of patients with metabolic disorders.

## 1 Introduction

The metabolic syndrome (MetS) (1) is a collection of metabolic abnormalities that include central obesity (Waist Circumference), triglycerides (TG), high-density lipoprotein cholesterol levels(HDL-C), hypertension, and glucose abnormalities. Its prevalence is increasing as society’s economic development and lifestyle change (2, 3). There is also a growing concern about the metabolic disease because it may increase the risk of not only all-cause mortality (4) but also increase cardiovascular disease, type 2 diabetes, and specific cancers (5, 6). Multiple interactions between environmental, metabolic, and genetic factors play a role in the pathogenesis of metabolic syndrome (7). Dietary changes are also thought to be a factor in the rising prevalence of metabolic disorders (8). Therefore, identifying relevant factors can help to prevent or reduce the occurrence of metabolic syndrome.

Iodine is an indispensable micronutrient for the human body. It regulates the growth and development of the body and tissue forms largely through the synthesis of thyroid hormones (9). Iodine is almost completely absorbed by the tiny intestine, while it is excreted mainly through the kidneys (10). The main sources of iodine in the diet are seafood (such as fish, crustaceans, and shellfish), eggs, milk, and products rich in iodine (11). The consumption of appropriate dietary iodine is essential for the maintenance of normal thyroid function. Iodine abnormalities (including iodine deficiency and iodine excess) are associated with goiters and abnormal thyroid function, which can increase the incidence of autoimmune thyroiditis and the risk of thyroid cancer (12, 13). Furthermore, iodine abnormalities can lead to developmental disorders, mental disorders, hearing loss, lower intelligence, and increased mortality in children (14, 15), as well as infertility, neurocognitive disorders, and narcolepsy in adults (16, 17).

Numerous studies have been conducted on iodine and thyroid-related diseases (18, 19). However, research on the effects of iodine on tissues and organs is limited. A few studies have shown that high or low levels of iodine in the body can directly or indirectly affect blood glucose, blood pressure, and lipid metabolism (20), but the results are not entirely consistent. Studies have confirmed that the prevalence of the metabolic syndrome is strongly related to regional economics and ethnicity, and varies by geographic location (21). The relationship between iodine and metabolic syndrome in American adults is currently unclear. The purpose of this study was to determine the impact of abnormal iodine consumption on metabolic syndrome in American adults. It will provide some reference to the effects of abnormal iodine on organs other than the thyroid gland.

## 2 Materials and methods

### 2.1 Study population

**Figure 1:**
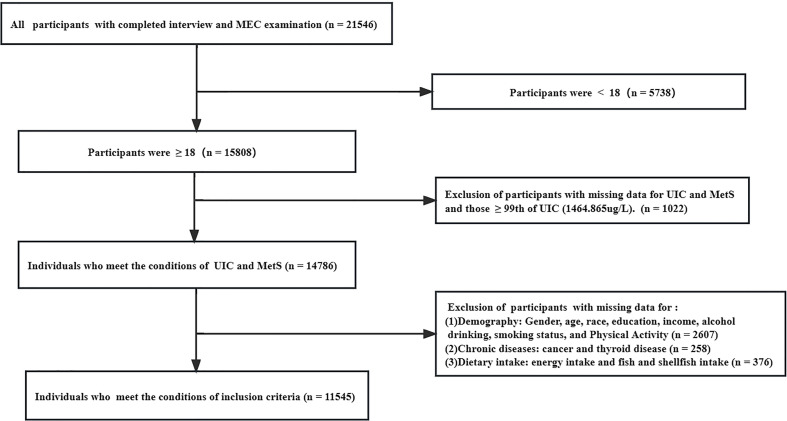
The Flow Chart of Inclusion and Exclusion in the study.

Data from six contiguous cycles of the National Health and Nutrition Examination Survey (NHANES) from 2007-2018. The data is publicly released on a two-year cycle and all of it used in the manuscript can be available on the website: https://wwwn.cdc.gov/nchs/nhanes/search/default.aspx. The NHANES is based on a stratified, multistage, and probability cluster designed and is mobilized by the National Center of Health Statistics of the Centers for Disease Control and Prevention to ensure sample representativeness (22). Participants were invited to stay at home or at a mobile examination center (MEC), where they were questioned about relevant demographic information, lifestyle, and diet and performed blood tests by professionally trained staff. The total sample size from 2007 to 2018 was 21,546 participants, of which 15,808 were 18 years and older. We excluded people with missing information on urinary iodine and metabolic syndrome as well as those with extreme values of iodine (> 99th percentile). Furthermore, we excluded data for missing demographics (1,211), relevant diet (376), lifestyle (1852), and related disease history (376), yielding a final sample of 11,545. Additional details about the study’s sampling and exclusion criteria are shown in **Figure 1**. The data was analyzed between November 2022 and January 2023. The study design strictly adheres to the guidelines of STROBE (Strengthening the Reporting of Observational Studies in Epidemiology) (23). Furthermore, the National Center for Health Statistics Research Ethics Review Board approved this study, and participants provided written informed consent. And Continuation of Protocol #2005-06, Protocol #2011-17, Continuation of Protocol #2011-17, and Protocol #2018-01 are the ethics approval numbers.

### 2.2 Diagnosis of metabolic syndrome

Metabolic syndrome (MetS) was defined according to the National Cholesterol Education Program/Adult Treatment Panel III criteria (NCEP-ATP III) (24), an update of the criteria proposed by the International Diabetes Federation and the World Health Organization, and was determined by meeting three to five of the following components (1): Central obesity: Waist Circumference (WC) ≥ 102 cm in men or ≥ 88 cm in women (2); hypertension: blood pressure ≥ 130/85 mmHg or hypertension history or treatment with antihypertensive medication (3); Diabetes: history of diabetes, glycosylated hemoglobin (HbA1c) > 6.5%, fasting glucose (mmol/L) ≥ 7.0, or medication used to treat diabetes (4); high TG: TG ≥ 150 mg/dL or pharmacological treatment of TG (5); low HDL-C: HDL-C < 40 mg/dL for men and < 50 mg/dL for women or using anti-lipid abnormalities. In NHANES, blood pressure was measured by a trained technician according to a standard protocol, using a manual mercury sphygmomanometer to measure the blood pressure of the participants, with every individual being measured three times (25). Since the first reading record was continuously higher than the second and third, while the latter two were closer. Therefore, if there are three measurements recorded, we would discard the first blood pressure reading and select the average of the second and third blood pressure readings for analysis. The average blood pressure was calculated by the following protocol (1): The diastolic reading with zero is not used to calculate the diastolic average (2); If all diastolic readings were zero, then the average would be zero (3); If only one blood pressure reading was obtained, that reading is average (4); If there is more than one blood pressure reading, the first reading is always excluded from the average.

### 2.3 Urinary iodine concentrations

Because almost all iodine ingested in the body is excreted in the urine (26), iodine is mostly present in the body as urine iodine. The World Health Organization (WHOs) also recommends that urine assessments be used to measure iodine levels in people (27). And Several studies have shown that urine iodine can be used as a proxy measure of dietary iodine content (28, 29). To sum up, urinary iodine concentration (UIC) was used indirectly as an indicator to estimate iodine consumption in the study, as there was a good correlation between iodine content in urine and intake. UIC as the most appropriate biochemical marker, which was determined by inductively coupled plasma dynamic reaction cell mass spectrometry (ICP-DRC-MS) (30). In NHANES, on-site urine samples were used to assess the nutritional status of iodine, while laboratory methods for the determination of UIC are publicly available (31). Urine iodine (µg/L) was divided into five groups from low to high according to the WHO guidelines: low UIC, **<** 100; normal UIC, 100-299; high UIC, 300-399; and very high, ≥ 400 (32).

### 2.4 Covariate assessments

Selecting covariates based on clinical experience, previous literature, and the statistical significance of reason. The questionnaire collected information on age, gender, race/ethnicity, education, income, alcohol consumption, smoking, physical activity, disease history, and medication history. Race/ethnicity was categorized as Mexican American, non-Hispanic white, non-Hispanic black, or other. Education levels were classified as below high school; high school grad/GED (General education development diploma) or Equivalent (33); above high school. Two categories of the annual family income were considered: “above $20000”, and “below $20000”. Participants were categorized as “mild”, “moderate”, “heavy”, and “no” based on the number of drinks per day he/she had drunk in 1 year. Participants who are “mild” were considered to be drinking alcohol ≤ 1 drink in women in 1 year or ≤ 2 drinks in men in 1 year; Participants who are “moderate” were considered to be drinking alcohol ≤ 2 drinks in women in 1 year or ≤ 3drinks in men in 1 year; Participants who are “heavy” were considered to be drinking alcohol ≥ 3 drinks of women in 1 year or ≥ 4drinks of man in 1 year; Participants who drank before last year but don’t drink now and those who never drank before are defined as “no” (34). Smoking status was defined as the numbers and timeline of cigarettes in life: “never” (smoked less than 100 cigarettes); “former” (smoked more than 100 cigarettes in life and smoke not at all now); “now” (smoked more than 100 cigarettes in life and smoke some days or every day now). Physical activity was measured based on the scores of the Metabolic Equivalent Task (MET) (35). Weekly as well as monthly exercise was converted to daily activity. The MET scores for vigorous-intensity work and vigorous recreational activities were calculated by multiplying by eight. You can get their MET score by multiplying cycling or walking, moderate-intensity activity, and moderate recreational activity by four. Cancer disease history was defined by a doctor’s diagnosis of cancer. The diagnosis of thyroid disease includes the use of thyroid medications and patients who have been diagnosed with thyroid disease by a physician. Other corresponding biochemical data such as Uric acid (umol/L), Triglyceride (mmol/L), HbA1c (%), HDL-C (mmol/L), LDL-C (mmol/L), Total cholesterol (mmol/L), and fasting glucose (mmol/L) were obtained from the blood Hemal Biochemistry file. Glomerular filtration rate (eGFR) (36) was calculated using the Chronic Kidney Disease Epidemiology Collaborative (CKD-EPI) formula,1:eGFR (mL/minute/1.73 m2) = 141*min(SCr/κ, 1) α*max(SCr/κ, 1)-1.209*0.993 age *1. 018 if female*1.159 If non-Hispanic black, where SCr is serum creatinine, κ is 0.7 for females and 0.9 for males, α is -0.329 for females and -0.411 for males, min indicates the minimum value of SCr/κ or 1, and max indicates the maximum value of SCr/κ or 1. Fasting glucose (37) tests were performed in the morning after a 9-hour fast, and after the initial venipuncture, participants were asked to drink a calibrated dose of TrutolTM (usually containing 75 g of glucose) and to perform a second venipuncture for 2 hours (plus or minus 15 minutes) after drinking TrutolTM for the OGTT. FT4 and TSH comes from the thyroid Profile, and the detailed instructions for specimen collection and handling are well documented in the NHANES Laboratory/Medical Technician Procedure Manual (38). For example, the TSH was obtained by Access HYPERsensitive human thyroid-stimulating hormone (hTSH) assay, which is a 3^rd^ generation, two-site immunoenzymatic (“sandwich”) assay.

### 2.5 Statistical analysis

We considered complex sampling designs and sample sizes during data analysis according to NHANES analysis guidelines (39). And the present data can represent a sample population of 67,771,194. Continuous variables were expressed as weighted means with standard deviation, whereas categorical variables are presented as cases (n) and percentage (%) categorical variables compared using Rao Scott’s χ2 test. One-way ANOVA was used to compare differences between groups. A multivariate logistic regression model was used to calculate the Odds ratio (OR) and 95% CI for the relationship between UIC and the prevalence of MetS, and the categorical normal group of UIC (≥ 100µg/L and < 300µg/L) was used as a reference. The crude model was not adjusted. Model 1 was adjusted for age and gender. Model 2 was adjusted for age, gender, education levels, race/ethnicity, smoking status, alcohol consumption, and physical activity. Model 3 was adjusted for Uric acid, eGFR, salt intake, energy intake, fish and shellfish intake, cancer, thyroid disorders, and the covariates of Model 2 besides. We investigated the continuous relationship between UIC (log10) and MetS by fitting a restricted cubic spline model at the 5th, 35th, 65th, and 95th percentiles of UIC (log10) (22). In addition, we further analyzed the relationship between UIC and the components of MetS. All analyzes were performed using the statistical software package R (http://www.r-project.org; version 4.2.2, The R Foundation) and Free Statistics software versions Statistics 1.7. And a two-sided *p-*value of less than 0.05 indicates a denoted statistically significant difference.

## 3 Results

### 3.1 Participant characteristics according to UIC

In this study, we selected nine continuous NHANES cycles (2007–2018) and focused on 11,545 adults with the completed interview, MEC examination, and laboratory examination in the US (≥18 years). Among the all participants in the study 5,740 (49.7%) females and 5,805 (50.3%) males were recruited, and a total of 3,200 participants had MetS. Based on the weighted analyzes, the mean UIC of total participants was 187.86 µg/L (IQR,184.58-191.14), which includes the recommended range by the WHO (IQR: 100-299µg/L), and those with education above high school accounted for 51.1%, and most of the participants were non-Hispanic white (44.5%). Participants with very high UIC whose value of glucose, triglyceride, and glucose of OGTT was higher and participants with low UIC were more likely to be female, non-Hispanic white, with higher annual household income, obesity, non-hypertensive, mild drinkers, nonsmokers, individuals who consume more fish or shellfish and with lower HbA1c (%). Hypertension, physical activity, systolic pressure, Sodium intake, and total cholesterol had no significant differences between the groups by UIC (*p* > 0.05). The baseline characteristics of the participants are summarized **in** **Table 1**.

**Table 1:** Baseline characteristics of participants with respect to urinary iodine.

Characteristics	Total	Low UIC (<100ug/L)	Normal UIC (100-299ug/L)	High UIC (300-399ug/L)	Very high UIC (≥400ug/L)	*P*-value
	11545	4064	5507	835	1139	
**UIC, (ug/L)**	187.86±3.28	60.14±0.51	175.96±1.03	343.55±1.51	627.15±7.87	< 0.001
**Age, (Years)**	47.12±0.30	45.26±0.37	47.67±0.40	49.88±0.74	49.58±0.67	< 0.001
**Gender, (%)**						< 0.001
Female	5740(49.7)	2218(55.5)	2631(48.4)	375(45.3)	516(45.2)	
Male	5805(50.3)	1846(44.5)	2876(51.6)	460(54.7)	623(54.8)	
**Ethnic, (%)**						0.004
Mexican American	1813(15.7)	586(7.7)	936(9.2)	129(7.8)	162(7.6)	
Non-Hispanic Black	2368(20.5)	889(10.9)	1137(11.1)	138 (8.0)	204 (9.3)	
Non-Hispanic White	5138(44.5)	1726(68.5)	2413(68.2)	422(74.9)	577(71.5)	
Other Race	2226(19.3)	863(12.9)	1021(11.4)	146 (9.3)	196(11.6)	
**Education levels, (%)**						0.02
Above high school	5940(51.5)	2182(62.3)	2794(58.4)	425(60.0)	539(57.4)	
Below high school	2875(24.9)	899(13.9)	1441(17.1)	213(16.3)	322(18.2)	
High School Grad/GED or Equivalent	2730(23.6)	983(23.8)	1272(24.5)	197(23.6)	278(24.4)	
**Income, (%)**						< 0.001
Above 20000$	9116(79)	3268(87.5)	4363(86.2)	651(86.5)	834(81.4)	
Below 20000$	2429(21)	796(12.5)	1144(13.8)	184(13.5)	305(18.6)	
**Alcohol drinking, (%)**						< 0.001
Heavy	2386(20.7)	883(22.6)	1114(22.0)	165(19.2)	224(22.7)	
Mild	3798(32.9)	1339(35.8)	1858(37.2)	266(36.6)	335(32.6)	
Moderate	1820(15.8)	735(20.9)	815(16.7)	123(16.1)	147(12.3)	
No	3541(30.7)	1107(20.7)	1720(24.1)	281(28.1)	433(32.5)	
**Smoking, (%)**						0.08
Former	2922(25.3)	961(24.9)	1420(25.3)	220(24.3)	321(28.6)	
Never	6221(53.9)	2194(53.3)	2982(55.1)	457(57.5)	588(53.4)	
Now	2402(20.8)	909(21.8)	1105(19.6)	158(18.3)	230(18.0)	
**Physical activity, (Met)**	567.6±14.3	532.6±20.8	596.2±22.5	612.0±49.3	529.7±29.8	0.11
**Energy, (kcal)**	2189.0±12.0	2157.4±21.6	2196.5±16.8	2305.3±36.4	2184.1±38.2	0.01
**Sodium intake, (mg/d)**	3579.0±22.8	3547.2±35.4	3603.0±32.3	3695.5±78.5	3492.5±66.1	0.22
**Fish and shellfish intake, (%)**						0.03
No	2290(19.8)	814(18.7)	1052(18.3)	158(18.7)	266(23.3)	
Yes	9255(80.2)	3250(81.3)	4455(81.7)	677(81.3)	873(76.7)	
**Waist, (cm)**	99.19±0.28	96.67±0.33	100.36±0.38	101.98±0.72	101.12±0.61	< 0.001
**OGTT glucose, (mmol/L)**	7.29±0.06	7.06±0.09	7.36±0.08	7.57±0.17	7.64±0.19	0.003
**Fasting glucose, (mmol/L)**	5.93±0.02	5.84±0.04	5.96±0.03	6.04±0.09	6.04±0.09	0.01
**HbA1c, (%)**	5.62±0.01	5.56±0.02	5.64±0.02	5.70±0.05	5.70±0.04	< 0.001
**eGFR**	94.89±0.45	97.19±0.61	94.06±0.47	91.54±0.95	92.65±0.85	< 0.001
**Uric acid, (umol/L)**	324.43±1.24	318.82±2.05	328.97±1.72	327.93±3.19	321.39±3.42	< 0.001
**TG, (mmol/L)**	2.73±0.03	2.64±0.05	2.73±0.05	2.89±0.12	2.96±0.10	0.03
**Total cholesterol, (mmol/L)**	5.02±0.02	5.04±0.02	5.02±0.02	4.96±0.05	4.95±0.05	0.23
**HDL, (mmol/L)**	1.38±0.01	1.43±0.01	1.36±0.01	1.32±0.02	1.31±0.02	< 0.001
**LDL, (mmol/L)**	2.42±0.01	2.43±0.03	2.44±0.02	2.34±0.04	2.32±0.04	0.02
**TSH, (mIU/L)**	1.95±0.04	1.98±0.10	1.91±0.06	1.98±0.04	1.91±0.06	0.661
**FT4, (ug/dL)**	7.98±0.03	8.00±0.09	8.00±0.04	7.96±0.04	7.99±0.07	0.824
**SBP, (mmHg)**	121.8±0.3	121.3±0.4	121.9±0.4	122.6±0.6	122.4±0.7	0.42
**DBP, (mmHg)**	70.98±0.22	71.56±0.32	70.62±0.24	70.26±0.60	71.01±0.45	0.02
**Cancer, (%)**						0.001
No	10401(90.1)	3754(91.6)	4928(89.5)	723(85.5)	996(89.2)	
Yes	1144(9.9)	310 (8.4)	579(10.5)	112(14.5)	143(10.8)	
**Thyroid disease, (%)**						0.002
No	10329(89.5)	3711(90.0)	4915(88.9)	729(86.5)	974(85.2)	
Yes	1216(10.5)	353(10.0)	592(11.1)	106(13.5)	165(14.8)	

UIC, urinary iodine concentration. MetS, metabolic syndrome; HbA1c, eGFR, Glomerular filtration rate; TG, triglyceride; HDL, high-density lipoprotein;

LDL, low-density lipoprotein; SBP, Systolic blood pressure; DBP, Diastolic blood pressure;

Data are presented as means (standard error), or weighted percentages as appropriate for the variable. Demographic and biochemical characteristics of the study population for Continuous normal variables were expressed as weighted mean ± standard deviation, One-way ANOVA was used to compare differences between groups. Categorical variables were expressed as frequencies and percentages and compared using Rao Scott’s χ2 test.

### 3.2 Prevalence of MetS and its components in different UIC groups

**Table 2** showed the prevalence of MetS was associated with UIC, and this relationship was found in both females and males. The overall prevalence of MetS was 27.7%. High waist circumference is the most common component of MetS, followed by hypertension, high TG levels, low HDL-C levels, and elevated glucose levels. When stratified by UIC, the prevalence of metabolic syndrome was 22.2%, 27.0%, 32.6 and 29.4% in the low-iodine, normal-iodine, and high-iodine, and very high-iodine groups, respectively. The prevalence of metabolic syndrome was significantly different among different UIC groups (*p* < 0.001). When analyzing the prevalence of each component of MetS in different UIC subgroups, we found that only hypertension was not statistically different from the UIC group (*p* = 0.99), while all other components were statistically different from the UIC subgroup (all *p* values < 0.05). In the gender subgroup, glucose abnormalities were statistically significant in different UIC groups in men (*p* = 0.006), however, this association was not seen in women (*p* = 0.125). Meanwhile, all other four groups were statistically different in the gender subgroup from the different UIC subgroups (p < 0.05).

**Table 2:** Prevalence of metabolic syndrome and its components in US adults with different urinary iodine concentration.

Variable		Prevalence (%) of Metabolic Syndrome and Its Components				
	Total	Normal UIC (100-299ug/L)	Low UIC (<100ug/L)	High UIC (300-399ug/L)	Very high UIC (≥400ug/L)	*P*-value
All participants						
**MetS**	3200 (27.7)	1627 (27.0)	962 (22.2)	262 (32.6)	349 (29.4)	< 0.001
**MetS.Diabetes**	3550 (30.7)	1831 (29.7)	1129 (25.3)	285 (32.8)	305 (24.7)	< 0.001
**MetS.low-HDL-C**	3571 (30.9)	1741 (30.3)	1151 (26.6)	293 (35.5)	386 (34.0)	< 0.001
**MetS.TG**	4248 (36.8)	2103 (37.1)	1298 (31.6)	350 (43.5)	497 (44.0)	< 0.001
**MetS.obesity**	6668 (57.8)	3284 (58.2)	2192 (53.6)	506 (61.1)	686 (59.5)	< 0.001
**MetS.hypertension**	2023 (17.5)	964 (15.9)	707 (16.1)	149 (16.2)	203 (15.6)	0.99
Female						
**MetS**	1724 (30.0)	857 (28.8)	566 (22.3)	118 (28.9)	183 (31.1)	< 0.001
**MetS.Diabetes**	1555 (27.1)	795 (26.7)	533 (20.8)	104 (27.4)	123 (19.8)	0.006
**MetS.low-HDL-C**	2102 (36.6)	982 (34.7)	751 (30.2)	147 (35.6)	222 (41.5)	0.001
**MetS.TG**	1856 (32.3)	908 (31.5)	598 (25.9)	141 (38.1)	209 (38.9)	< 0.001
**MetS.obesity**	3994 (69.6)	1896 (68.8)	1438 (61.2)	275 (71.6)	385 (71.7)	< 0.001
**MetS.hypertension**	939 (16.4)	447 (16.1)	351 (14.5)	65 (16.1)	76 (12.3)	0.322
Male						
**MetS**	1476 (25.4)	770 (25.4)	396 (21.9)	144 (35.6)	166 (28.0)	< 0.001
**MetS.Diabetes**	1995 (34.4)	1036 (32.5)	596 (30.8)	181 (37.2)	182 (28.7)	0.125
**MetS.low-HDL-C**	1469 (25.3)	759 (26.1)	400 (22.3)	146 (35.4)	164 (27.7)	< 0.001
**MetS.TG**	2392 (41.2)	1195 (42.3)	700 (38.7)	209 (48.0)	288 (48.2)	0.004
**MetS.obesity**	2676 (46.1)	1388 (48.0)	754 (44.0)	231 (52.4)	301 (49.5)	0.05
**MetS.hypertension**	1084 (18.7)	517 (15.9)	356 (18.0)	84 (16.3)	127 (18.3)	0.484

UIC, urinary iodine concentration. MetS, metabolic syndrome,

MetS.Diabetes, History of diabetes, Glycosylated hemoglobin (HbA1c) > 6.5%, fasting glucose (mmol/L) ≥ 7.0, or medication used to treat diabetes;

MetS.obesity, Waist Circumference (WC) ≥ 102 cm in men or ≥ 88 cm in women;

MetS.hypertension, blood pressure ≥ 130/85 mmHg or hypertension history or treatment with antihypertensive medication;

MetS.low-HDL-C, HDL-C < 40 mg/dL for men and < 50 mg/dL for women or use of anti-lipid abnormalities;

MetS.TG, TG ≥ 150 mg/dL, or pharmacological treatment of TG. The prevalence of metabolic syndrome components according to the urinary iodine concentration was compared using Rao Scott’s χ2 test.

### 3.3 The relationship between UIC and the risk of MetS and components

**Figure 2:**
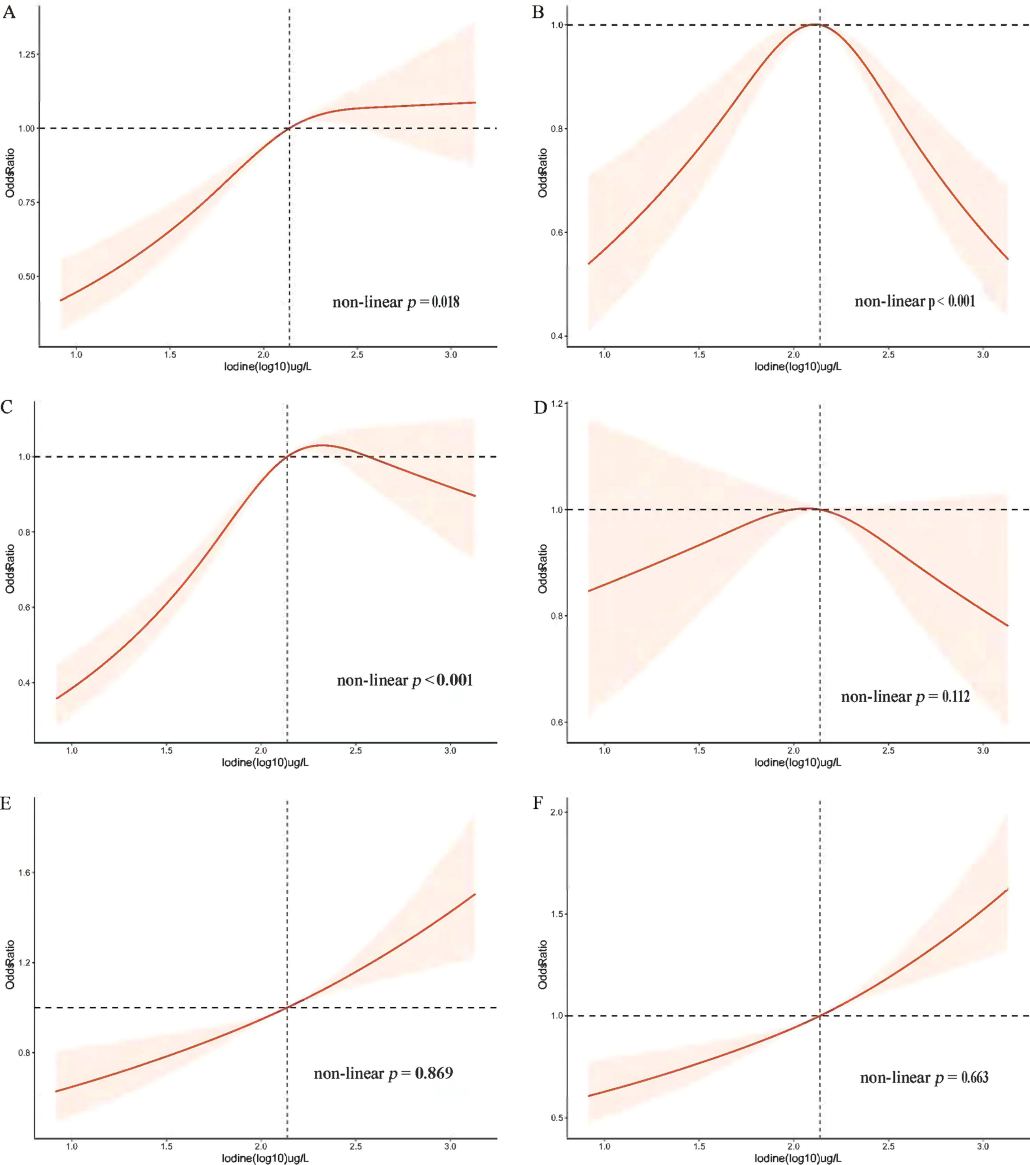
Restricted cubic spline plot of the association between UIC and MetS and components in participants of American adults (age ≥ 18). **(A)** Prevalence of metabolic syndrome (MetS); **(B)** Prevalence of diabetes mellitus (Diabetes); **(C)** Prevalence of obesity; **(D)** Prevalence of hypertension; **(E)** Prevalence of low High-density lipoprotein (low-HDL-C); **(F)** Prevalence of triglycerides (TG). Adjusted for age, sex, race/ethnicity, education, annual family income, smoking status, alcohol intake, physical activity, thyroid problems, cancer, energy intake, fish or shellfish intake, sodium intake, eGFR (Glomerular filtration rate), TSH, and FT4.

The OR and corresponding 95% CI of the risk for MetS according to UIC (log10) and four UIC groups are summarized in **Table 3**. When UIC was a continuous value, a multivariate regression model was used to adjust for other possible confounding factors, including demographic factors, chronic illness, lifestyle habits, dietary factors, and thyroid hormones. We found that with each log-unit increase in UIC level, there was a corresponding 50% increase in the probability of developing MetS (OR, 1.50; 95% CI, 1.275-1.756; *p* < 0.001). The same trend was observed in the category of low UIC group (< 100µg/L) has a protective effect on MetS (OR, 0.82; 95%CI, 0.708-0.946; *p* = 0.007) and high UIC group (300-399µg/L) has an adverse effect on MetS (OR, 1.25; 95%CI, 1.016-1.539; *p* = 0.035) compared to the normal group of UIC, adjusting for all relevant covariates in **Table 3**. The relationship between the risk of other MetS components and UIC was similarly validated. Furthermore, restricted cubic splines showed the relationship between UIC and MetS and components of MetS **in** **Figure 2**. And a *p-value* less than 0.05 shows non-linearity, while a *p-value* greater than 0.05 represents a linear relationship.

**Table 3:** Multiple logistic regression between UIC and MetS and its components.

Variables	N (%)	Crude model		Model 1		Model 2		Model 3	
		OR (95%CI)	*p*	OR (95%CI)	*p*	OR (95%CI)	*p*	OR (95%CI)	*p*
MetS									
**UIC (log10)**		1.69(1.441,1.982)	<0.001*	1.54(1.301,1.816)	<0.001*	1.50(1.272,1.757)	<0.001*	1.50(1.275,1.756)	<0.001*
Classified UIC									
normal UIC	1627(48.23)	ref		ref		ref		ref	
low UIC	962(31.68)	0.77(0.665,0.891)	<0.001*	0.81(0.699,0.941)	0.006*	0.82(0.706,0.949)	0.009*	0.82(0.708,0.946)	0.007*
high UIC	262(9.48)	1.31(1.069,1.600)	0.01*	1.26(1.018,1.547)	0.033*	1.26(1.020,1.555)	0.033*	1.25(1.016,1.539)	0.035*
very high UIC	349(10.61)	1.13(0.933,1.358)	0.213	1.08(0.888,1.314)	0.435	1.04(0.858,1.263)	0.681	1.05(0.862,1.270)	0.644
*p for trend*			0.222		0.39		0.531		0.515
MetS.Diabetes									
normal UIC	1831(49.24)	ref		ref		ref		ref	
low UIC	1129(33.61)	0.80(0.704,0.916)	0.001*	0.90(0.791,1.026)	0.114	0.91(0.798,1.032)	0.138	0.90(0.791,1.022)	0.103
high UIC	285(8.86)	1.16(0.909,1.470)	0.234	1.06(0.814,1.382)	0.659	1.08(0.828,1.407)	0.57	1.08(0.830,1.407)	0.560
very high UIC	305(8.28)	0.78(0.622,0.972)	0.027*	0.70(0.555,0.869)	0.002*	0.69(0.549,0.860)	0.001*	0.68(0.542,0.855)	0.001*
*p for trend*			0.063		0.009*		0.01*		0.008*
MetS.obesity									
normal UIC	3265(47.12)	ref		ref		ref		ref	
low UIC	2189(34.93)	0.84(0.749,0.946)	0.004*	0.84(0.742,0.947)	0.005*	0.85(0.749,0.954)	0.007*	0.83(0.731,0.945)	0.005*
high UIC	502(8.03)	1.12(0.919,1.357)	0.262	1.08(0.881,1.329)	0.448	1.09(0.879,1.356)	0.422	1.11(0.885,1.390)	0.364
very high UIC	691(9.92)	1.11(0.933,1.309)	0.246	1.08(0.910,1.281)	0.376	1.05(0.879,1.252)	0.592	1.01(0.837,1.204)	0.970
*p for trend*			0.454		0.693		0.852		0.856
MetS.hypertension									
normal UIC	964(46.18)	ref		ref		ref		ref	
low UIC	707(37.13)	1.01(0.857,1.180)	0.947	1.12(0.952,1.319)	0.169	1.12(0.948,1.322)	0.181	1.13(0.953,1.329)	0.162
high UIC	149(7.61)	1.01(0.782,1.315)	0.915	0.94(0.718,1.224)	0.631	0.96(0.731,1.252)	0.746	0.95(0.726,1.237)	0.688
very high UIC	203(9.08)	0.97(0.775,1.211)	0.775	0.90(0.706,1.135)	0.357	0.91(0.719,1.154)	0.435	0.92(0.725,1.166)	0.481
*p for trend*			0.876		0.532		0.654		0.683
MetS.low-HDL-C									
normal UIC	1741(47.09)	ref		ref		ref		ref	
low UIC	1151(33.23)	0.84(0.758,0.928)	<0.001*	0.80(0.723,0.887)	<0.001*	0.81(0.729,0.895)	<0.001*	0.81(0.732,0.894)	<0.001*
high UIC	293(9.00)	1.27(1.020,1.578)	0.033*	1.31(1.041,1.636)	0.021*	1.31(1.046,1.634)	0.019*	1.30(1.043,1.630)	0.020*
very high UIC	386(10.68)	1.19(1.002,1.401)	0.047*	1.22(1.030,1.436)	0.021*	1.17(0.990,1.392)	0.065	1.18(0.985,1.410)	0.072
*p for trend*			0.049*		0.033*		0.061		0.064
MetS.TG									
normal UIC	2103(47.30)	ref		ref		ref		ref	
low UIC	1298(32.31)	0.79(0.691,0.893)	<0.001*	0.84(0.739,0.960)	0.011*	0.84(0.738,0.959)	0.011*	0.85(0.746,0.965)	0.013*
high UIC	350(9.05)	1.31(1.069,1.604)	0.01*	1.25(1.016,1.547)	0.035*	1.25(1.013,1.537)	0.038*	1.24(1.002,1.533)	0.048*
very high UIC	497(11.35)	1.33(1.137,1.565)	<0.001*	1.28(1.083,1.522)	0.004*	1.26(1.068,1.492)	0.007*	1.26(1.082,1.503)	0.004*
*p for trend*			0.006*		0.018*		0.024*		0.018*

UIC, urinary iodine concentration; MetS, metabolic syndromes; HDL, high-density lipoprotein; TG, triglyceride.

MetS.Diabetes, History of diabetes, Glycosylated hemoglobin (HbA1c) > 6.5%, fasting glucose (mmol/L) ≥ 7.0, or medication used to treat diabetes;

MetS.obesity, Waist Circumference (WC) ≥ 102 cm in men or ≥ 88 cm in women;

MetS.hypertension, blood pressure ≥ 130/85 mmHg or hypertension history or treatment with antihypertensive medication;

MetS.low-HDL-C, HDL-C < 40 mg/dL for men and < 50 mg/dL for women or use of anti-lipid abnormalities;

MetS.TG, TG ≥ 150 mg/dL or pharmacological treatment of TG.

Data are expressed as weighted percentages. OR and 95% CI for risk of metabolic syndrome and its components were estimated using complex samples logistic regression. * represents p < 0.05.

Normal UIC: < 100ug/L; Low UIC: 100-299ug/L; High UIC: 300-399ug/L; Very high UIC: ≥ 400ug/L.

Crude Model: not adjusted;

Model 1: Adjusted for age, sex;

Model 2: Adjusted for age, sex, race/ethnicity, education, the annual family income, smoking status, alcohol intake, physical activity,

Model 3: Adjusted for age, sex, race/ethnicity, education, the annual family income, smoking status, alcohol intake, physical activity, thyroid problems, cancer, energy intake, fish or shellfish intake, sodium intake, eGFR, TSH, and FT4.

The association between blood glucose abnormalities and UIC (log10) showed an inverted u-shaped curve with a maximum point of 2.14 µg/L and a non-linear p < 0.001 (**Figure 2**). **Figure 2** demonstrated an approximately inverted U-shaped relationship between obesity and UIC (log10) (non-linear p < 0.001). **Table 3** showed that the very high UIC group (≥ 400 µg/L) was associated with a lower occurrence of glucose abnormalities compared to the normal group UIC (OR, 0.68; 95%CI, 0.542-0.855; *p* = 0.001), but the incidence of reducing obesity was associated with lower UIC (OR, 0.83; 95%CI, 0.731-0.945; *p* = 0.005). In addition, we further analyzed the relationship between BMI and UIC in **Table S1**. We found a positive correlation between urinary iodine and BMI (Coefficient,1.97; 95% CI: 1.591 2.355; p < 0.001). And BMI was lower in participants with low UIC compared to those with the normal UIC group (Coefficient, -1.29; 95% CI: -1.644 to -0.936; p < 0.001). Because there is co-linearity between BMI and waist circumference (40), we did not adjust BMI in the process of data analysis. The results in **Table S1** once again verify the accuracy of the relationship between obesity and UIC, which is the component of MetS in **Table 3**. At the same time, Lower UIC favored lower HDL-C (OR, 0.81; 95%CI, 0.732-0.894; *p* < 0.001), whereas higher UIC (300-399ug/L) increased the incidence of HDL-C (OR, 1.30; 95%CI, 1.043-1.630; *p* = 0.020), although the 95% CI in the very high UIC group spanned 1. Furthermore, Low UIC (< 100µg/L) was associated with lower TG levels but the incidence of TG levels was higher when UIC was ≥ 300µg/L. However, the occurrence of hypertension was not associated with UIC both higher and lower UIC compared to normal UIC. There was a linear relationship between hypertension, HDL, TG, and UIC (log10) with their *p* of non-linear were 0.112,0.869, and 0.663 in **Figure 2**, respectively.

**Figure 3:**
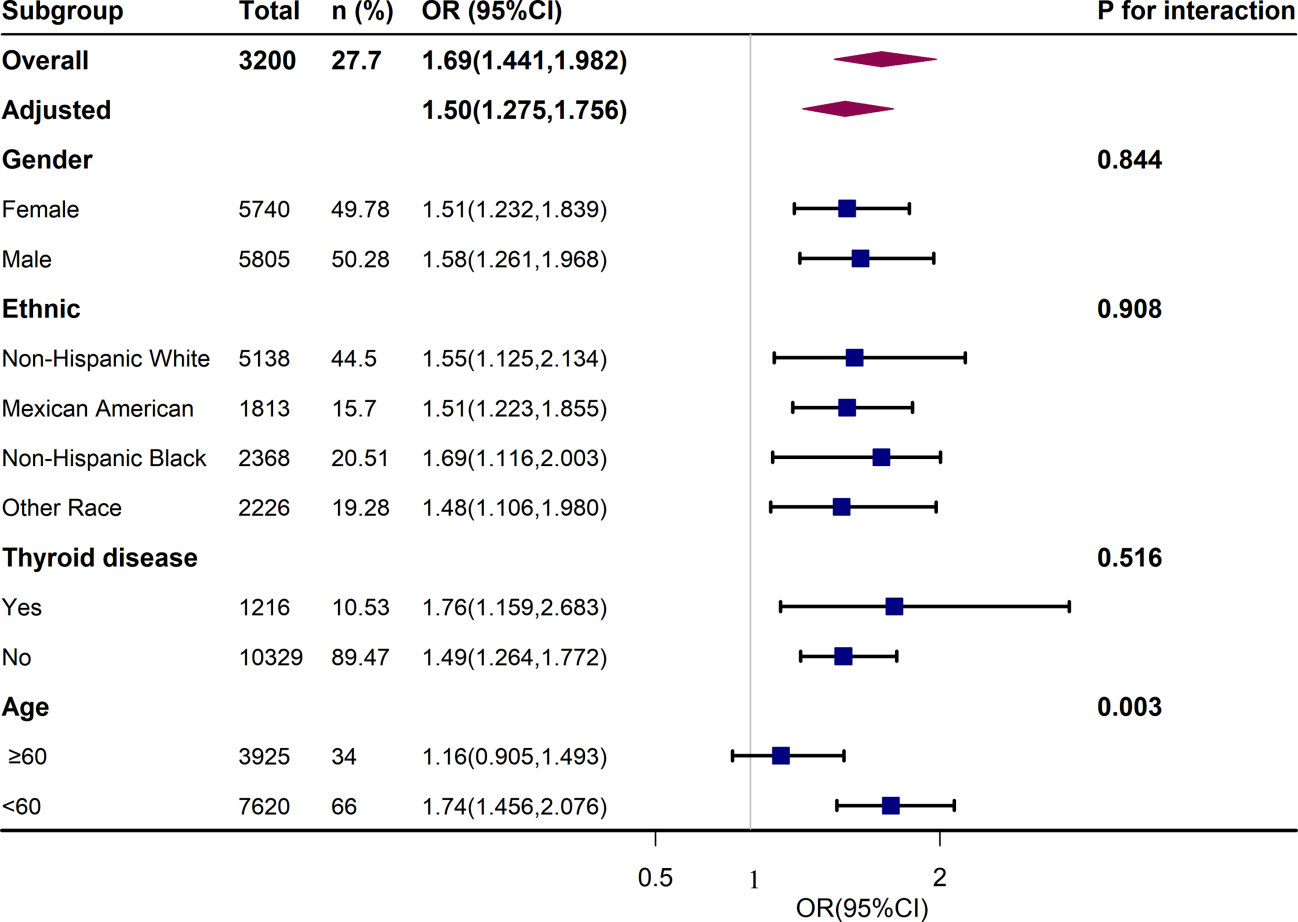
Association Between UIC (log10) and metabolic syndrome (MetS). Each stratification was adjusted for age, sex, race/ethnicity, education, annual family income, smoking status, alcohol intake, physical activity, thyroid problems, cancer, energy intake, fish or shellfish intake, sodium intake, eGFR (Glomerular filtration rate), TSH, and FT4.

We performed further sensitivity analysis. After excluding 10.5% of patients with thyroid disease, we also analyzed the relationship between different UIC levels and the metabolic syndrome and its components, as shown in **Table S2**. We found that low UIC (<100ug/L) was associated with a higher incidence of hypertension, and there was no statistical difference between UIC (<100ug/L) and metabolic syndrome. Additional results were consistent with those already analyzed in patients without thyroid exclusion in **Table 3**. In addition, the results of the subgroup analysis of age, sex, race, and thyroid disease are presented in **Figure 3**. After adjusting for education, annual family income, smoking status, alcohol intake, physical activity, cancer, energy intake, fish or shellfish intake, sodium intake, eGFR, TSH, and FT4, we found an interaction in the age stratification (*p* = 0.003), with a positive association between UIC (log10) (continuous variable) and MetS when the age was less than 60, while there was no statistically significant association in the participants with age ≥ 60. However, there was no interaction between UIC and MetS in the other subgroups as shown in **Table S3**.

## 4 Discussion

This study evaluated a nationally representative sample of American adults and discovered a positive correlation between UIC and the risk of MetS, though after controlling for potentially significant confounders. We also discovered a non-linear relationship between UIC and MetS in the fully adjusted model (non-linear *p* = 0.017). We investigated the prevalence of MetS and its components in participants aged ≥ 18 years in this cross-sectional study based on US NHANES data, as well as the relationship between iodine status and MetS and their components. We not only demonstrated the relationship between UIC (log10) and MetS and their components, but we also looked into it in different UIC strata. We found that UIC of < 100µg/L was significantly associated with obesity and, low HDL-C, MetS, and TG. There is also a significant correlation between UIC and MetS, low HDL-C, and TG when UIC is between 300 and 399 ug/L. And the UIC of ≥ 400µg/L was linked to fewer glucose abnormalities. However, after adjusting for confounders, there was no significant association between each UIC group and hypertension compared to the normal group.

In the sensitivity analysis, we found that low urinary iodine (UIC<100ug/L) was associated with a higher incidence of hypertension, and there was no statistical difference between urinary iodine (UIC<100ug/L) and metabolic syndrome. Other results were consistent with those already analyzed in patients without thyroid exclusion. According to the data presented above, high UIC may be potentially protective against the development of blood glucose abnormalities and have a positive correlation with low HDL-C levels, TG, and MetS. However, low iodine UIC is potentially protective against low HDL-C levels, TG, and obesity. As a result, the detection of iodine has a crucial role in metabolic disorders.

To our knowledge, studies have investigated the relationship between iodine and metabolism-related diseases and support our results. A study of the relationship between iodine and MetS in Central American children and parents confirmed that high iodine concentration increases the incidence of MetS in adults (41). In the study, we also found that lower UIC was associated with a lower incidence of metabolic syndrome, whereas this association was not found after excluding participants with thyroid disease. A cross-sectional study of epidemiology in China indicated that the association between UIC and MetS was not present in iodine deficiency (<100µg/L) in non-thyroid disease populations (42). Similarly, Kim et al. found no association between iodine intake and MetS risk in male Korean adults ≥ 19 years of participants with normal thyroid function (43). In the present study, UIC was negatively associated with the prevalence of diabetes, TG levels, and low HDL levels at ≥ 400 µg/L, ≥ 300µg/L, and < 100 µg/L, respectively. A nationwide cross-sectional epidemiological study from China discovered a link between UIC ≥ 300μg/L and dyslipidemia, which also supports our results (42). Following that, a randomized controlled trial of iodine supplementation in overweight women reported that iodine supplementation reduced the incidence of hypercholesterolemia (44). One study reported that mild iodine deficiency (100-150μg/L) was associated with an increased risk of gestational diabetes (45). Larsson et al. observed that adequate iodide levels improved insulin secretory function in isolated pancreatic islets during glucose-stimulated insulin secretion (46). we also found that low urinary iodine (UIC<100ug/L) was associated with a higher incidence of hypertension in participants without thyroid disorders exclusion, but there was no significant correlation between each UIC group and hypertension compared to the normal group in all participants without excluding those who were thyroid disease. Next, an Indian cross-sectional study (47) showed that iodine levels were negatively correlated with age and SBP (42). A study (31) of US adults from 2010 found that current hypertension or a history of hypertension was also not significantly associated with iodine deficiency or high iodine status in men and women compared to those who did not have hypertension. Furthermore, a cross-sectional study (42) from TIDE data showed that UIC <100 μg/L was associated with hypertension (OR, 1.097;95%CI:1.035-1.162) in participants without thyroid disorder. Moreover, our study shows that UIC is positively associated with obesity and BMI. Pablo and his colleague’s study of 10 public elementary schools in 50 cities in the Mexican state of Queretaro showed the same positive relationship between the median UIC and the median body mass index (BMI) in each school (48). And Farebrother et al. recently studied the iodine nutritional status of multiracial obese pregnant women from inner-city areas in the United Kingdom and found that lower iodine status was associated with lower birth weight (49).

However, some studies contradict our findings. Several clinical trials have reported a negative association between high UIC and dyslipidemia and MetS. According to the Riyadh Cohort Study (50) found that UIC was significantly lower in type 2 diabetic patients than in healthy controls. The survey, which Lee and his colleagues completed in 2016, confirmed that individuals in the lowest UIC 10th percentile had a higher probability of dyslipidemia than adults above the 10th percentile and that UIC was protective against low HDL-C in the United States (51). In a Chinese study on iodine and dyslipidemia in drinking water, HDL-C was found to be negatively correlated with iodine in water in the excess iodine group (52). Subsequently, the TIDE study (42) confirmed that a slightly higher UIC (300-399µg/L) had a protective effect on reducing MetS, which is inconsistent with our results. In our nationally representative epidemiological cross-sectional survey, we discovered that slightly higher UIC (≥ 300µg/L and < 400µg/L) was associated with higher levels of TG and a higher prevalence of MetS when compared to normal UIC. An animal test discovered that TG levels in male mice and TG levels in female mice in the excess iodine group were much lower than in the normal iodine group (53). A study (20) from Shanxi verified that excessive iodine intake may result in elevated blood glucose and blood pressure. Moreover, a Korean multi-rural community cohort study (47) found no significant relationship between dietary iodine, seaweed consumption, and waist circumference in postmenopausal women. In contrast, a review (54) reported that low UIC increased the prevalence of obesity. These differences can be explained by a variety of reasons. First, it is possible that the iodine was collected in different ways, for example, some studies used on-site urine samples, others conducted 24-hour collections, and still, others used aqueous iodine concentrations; in addition, there were also subtle differences in the definitions of the studies’ outcomes. Furthermore, differences in ethnicity, age, and national populations, as well as differences in salt intake, may also account for the disparities.

The physiopathological mechanisms and explanations of iodine and metabolic disorders are currently unclear and rare, but the following points may help to explain them. First, the potential effect of excess iodine on MetS and components may be due to the interaction of nutrients with thyroid hormones (55). Although iodine intake and status are not directly related to thyroid hormone levels, both severe iodine deficiency and severe iodine excess can lead to hypothyroidism (56). TSH has been found to be positively correlated with triglycerides (57) and the balance of insulin resistance (58). Second, chronic inflammatory states and oxidative stress appear to be central to the pathophysiology of MetS. Excess iodine puts cells in a state of high oxidative stress, which predisposes them to organ damage and is detrimental to disease recovery (59). Moreover, iodine functions as both a pro-oxidant and an antioxidant (60, 61), and it can balance oxidative homeostasis at the physiological and molecular levels of the cell (62). For example, iodine acts as a free radical and can prevent the reaction of iodinated tyrosine, histidine, and some polyunsaturated fatty acids from reacting with oxygen radical double bonds (63). As a result, thyroid function is limited when iodine is insufficient, thus reducing the rate of metabolism in order to reduce the risk of MetS and dyslipidemia. Similarly, when iodine is higher, thyroid hormones are produced more, leading to increased metabolism to further increase the risk of MetS and dyslipidemia. The association between excess UIC and reduced incidence of diabetes could be explained by the anti-inflammatory and antioxidant effects of iodine, as well as the decreased rate of liver glycogen synthesis due to excess iodine-induced hypothyroidism. Low urinary iodine may lead to decreased thyroid hormone levels, which in turn affect the function of the cardiovascular system and increase the incidence of hypertension. In addition, low urinary iodine may also affect kidney function, resulting in an imbalance of water and sodium balance in the body, which may also indirectly lead to the occurrence of high blood pressure. Thus, low UIC is associated with an increased risk of hypertension in participants with normal thyroid function. When iodine is deficient, insufficient thyroid hormone production reduces the body’s demand for energy, which may influence participants’ behavior, such as reducing energy intake, resulting in decreased fat synthesis, and thus reducing the incidence of obesity.

This study has several advantages. Firstly, this study includes data accumulated in the NHANES database, which covers states across the United States, visiting 15 of these counties each year. Therefore, the sample size was adequate. Secondly, we adjusted for diet, lifestyle habits, demographic information, thyroid medications, and thyroid disease in the full model. However, there are also some limitations in this study. Firstly, this is a cross-sectional survey, and the causal relationship between UIC and MetS and their components cannot be established. Secondly, we adjusted for as many potential confounders as possible, but it is still possible that some factors were not included. For example, seaweeds and sea vegetables are important sources of iodine. And iodine content is also reported to vary widely among seaweed species (64), which are rarely eaten in the United States, making estimates using NHANES data impractical. Therefore, we cannot exclude the effect of seaweed and seaweed on UIC. Thirdly, we excluded adolescents under the age of 18 and thus cannot represent the entire US population, but only US adults. We will further investigate this association in the future.

## 5 Conclusions

In conclusion, excess iodine is associated with lowered hyperglycemia. A slightly higher UIC is associated with increased MetS and TG. However, low iodine may be associated with a reduced prevalence of metabolic disorders and their associated diseases, including MetS, low HDL-C, TG, and central obesity. While our study adds to the existing evidence on UIC and metabolic syndrome and its components, there has been little research on this relationship, which warrants further investigation by related studies.

## Data availability statement

The datasets presented in this study can be found in online repositories. The names of the repository/repositories and accession number(s) can be found below: https://wwwn.cdc.gov/nchs/nhanes/search/default.aspx.

## Ethics statement

This study was supported by the National Center for Health Statistics Research Ethics Review Board, and the ethics approval number is Continuation of Protocol #2005-06, Protocol #2011-17, Continuation of Protocol #2011-17, and Protocol #2018-01. You can find it at this website: https://www.cdc.gov/nchs/nhanes/irba98.htm. This study is an analysis of the publicly available NHANES data. Informed consent was obtained from NHANES participants by the National Center for Health Statistics Research Ethics Review Board.

## Author contributions

XS and LY contributed to the conception and design, acquisition and drafting of the manuscript or critical revision for important intellectual content. Y-YL and X-HZ contributed to interpretation of the data and analysis. PC, J-FH and LJ contributed to the conception and design and reviewing of the manuscript or critical revision for important intellectual content. All authors approved the final version, and agree to be accountable for all aspects of the work.

## References

[1] He J Lai Y Yang J Yao Y Li Y Teng W. The relationship between thyroid function and metabolic syndrome and its components: a cross-sectional study in a Chinese population. Front Endocrinol (Lausanne) (2021) 12:661160. doi: 10.3389/fendo.2021.661160 33868183 PMC8044548

[2] Aguilar M Bhuket T Torres S Liu B Wong RJ. Prevalence of the metabolic syndrome in the united states, 2003-2012. JAMA (2015) 313(19):1973–4. doi: 10.1001/jama.2015.4260 25988468

[3] de Siqueira Valadares LT de Souza LSB Salgado Júnior VA de Freitas Bonomo L de Macedo LR Silva M. Prevalence of metabolic syndrome in Brazilian adults in the last 10 years: a systematic review and meta-analysis. BMC Public Health (2022) 22(1):327. doi: 10.1186/s12889-022-12753-5 35172790 PMC8848905

[4] Malik S Wong ND Franklin SS Kamath TV L'Italien GJ Pio JR. Impact of the metabolic syndrome on mortality from coronary heart disease, cardiovascular disease, and all causes in united states adults. Circulation (2004) 110(10):1245–50. doi: 10.1161/01.CIR.0000140677.20606.0E 15326067

[5] Esposito K Chiodini P Colao A Lenzi A Giugliano D. Metabolic syndrome and risk of cancer: a systematic review and meta-analysis. Diabetes Care (2012) 35(11):2402–11. doi: 10.2337/dc12-0336 PMC3476894 23093685

[6] Taylor JY Kraja AT de Las Fuentes L Stanfill AG Clark A Cashion A. An overview of the genomics of metabolic syndrome. J Nurs Scholarsh (2013) 45(1):52–9. doi: 10.1111/j.1547-5069.2012.01484.x PMC3594572 23368731

[7] Lu J Wang L Li M Xu Y Jiang Y Wang W. Metabolic syndrome among adults in China: the 2010 China noncommunicable disease surveillance. J Clin Endocrinol Metab (2017) 102(2):507–15. doi: 10.1210/jc.2016-2477 27898293

[8] Fenwick PH Jeejeebhoy K Dhaliwal R Royall D Brauer P Tremblay A. Lifestyle genomics and the metabolic syndrome: a review of genetic variants that influence response to diet and exercise interventions. Crit Rev Food Sci Nutr (2019) 59(13):2028–39. doi: 10.1080/10408398.2018.1437022 29400991

[9] Andersson M Braegger CP. The role of iodine for thyroid function in lactating women and infants. Endocr Rev (2022) 43(3):469–506. doi: 10.1210/endrev/bnab029 35552681 PMC9113141

[10] Perry WF Hughes JFS. The urinary excretion and thyroid uptake of iodine in renal disease. J Clin Invest (1952) 31(5):457–63. doi: 10.1172/JCI102630 PMC436440 14927736

[11] Krela-Kaźmierczak I Czarnywojtek A Skoracka K Rychter AM Ratajczak AE Szymczak-Tomczak A. Is there an ideal diet to protect against iodine deficiency? Nutrients (2021) 13(2):513. doi: 10.3390/nu13020513 PMC7914421 33557336

[12] Zimmermann MB Boelaert K. Iodine deficiency and thyroid disorders. Lancet Diabetes Endocrinol (2015) 3(4):286–95. doi: 10.1016/S2213-8587(14)70225-6 25591468

[13] Ehlers M Schott M. Hashimoto's thyroiditis and papillary thyroid cancer: are they immunologically linked? Trends Endocrinol Metab (2014) 25(12):656–64. doi: 10.1016/j.tem.2014.09.001 25306886

[14] Bleichrodt N Drenth PJ Querido A. Effects of iodine deficiency on mental and psychomotor abilities. Am J Phys Anthropol (1980) 53(1):55–67. doi: 10.1002/ajpa.1330530110 7416250

[15] Millon-Ramirez C García-Fuentes E Soriguer F. Iodine deficiency and hearing impairment. JAMA Otolaryngol Head Neck Surg (2019) 145(1):94–5. doi: 10.1001/jamaoto.2018.2755 30383149

[16] Hollowell JG Staehling NW Hannon WH Flanders DW Gunter EW Maberly GF. Iodine nutrition in the united states. trends and public health implications: iodine excretion data from national health and nutrition examination surveys I and III (1971-1974 and 1988-1994). J Clin Endocrinol Metab (1998) 83(10):3401–8. doi: 10.1210/jcem.83.10.5168 9768638

[17] Dunn JT Delange F. Damaged reproduction: the most important consequence of iodine deficiency. J Clin Endocrinol Metab (2001) 86(6):2360–3. doi: 10.1210/jcem.86.6.7611 11397823

[18] Chen W Zhang Y Hao Y Wang W Tan L Bian J. Adverse effects on thyroid of Chinese children exposed to long-term iodine excess: optimal and safe tolerable upper intake levels of iodine for 7- to 14-y-old children. Am J Clin Nutr (2018) 107(5):780–8. doi: 10.1093/ajcn/nqy011 29722836

[19] Farebrother J Zimmermann MB Andersson M. Excess iodine intake: sources, assessment, and effects on thyroid function. Ann N Y Acad Sci (2019) 1446(1):44–65. doi: 10.1111/nyas.14041 30891786

[20] Liu J Liu L Jia Q Zhang X Jin X Shen H. Effects of excessive iodine intake on blood glucose, blood pressure, and blood lipids in adults. Biol Trace Elem Res (2019) 192(2):136–44. doi: 10.1007/s12011-019-01668-9 30798477

[21] Salsberry PJ Corwin E Reagan PB. A complex web of risks for metabolic syndrome: race/ethnicity, economics, and gender. Am J Prev Med (2007) 33(2):114–20. doi: 10.1016/j.amepre.2007.03.017 17673098

[22] Statistics CfDCaPNCfH.December 2018 NHANES survey methods and analytic Guidelines, 2011-2016. Available at: https://wwwn.cdc.gov/nchs/nhanes/analyticguidelines.aspx#sample-design (Accessed January 18, 2023).

[23] Vandenbroucke JP von Elm E Altman DG Gøtzsche PC Mulrow CD Pocock SJ. Strengthening the reporting of observational studies in epidemiology (STROBE): explanation and elaboration. Epidemiol (Cambridge Mass) (2007) 18(6):805–35. doi: 10.1097/EDE.0b013e3181577511 18049195

[24] Expert Panel on Detection, Evaluation, and Treatment of High Blood Cholesterol in Adults. Executive Summary of the Third Report of the National Cholesterol Education Program (NCEP) Expert Panel on Detection, Evaluation, and Treatment of High Blood Cholesterol in Adults (Adult Treatment Panel III). JAMA (2001) 285(19):2486–97. doi: 10.1001/jama.285.19.2486 11368702

[25] Ong KL Cheung BMY Man YB Lau CP Lam KSL. Prevalence, awareness, treatment, and control of hypertension among united states adults 1999-2004. Hypertension (2007) 49(1):69–75. doi: 10.1161/01.HYP.0000252676.46043.18 17159087

[26] Vejbjerg P Knudsen N Perrild H Laurberg P Andersen S Rasmussen LB. Estimation of iodine intake from various urinary iodine measurements in population studies. Thyroid (2009) 19(11):1281–6. doi: 10.1089/thy.2009.0094 19888863

[27] Peniamina R Skeaff S Haszard JJ McLean R. Comparison of 24-h diet records, 24-h urine, and duplicate diets for estimating dietary intakes of potassium, sodium, and iodine in children. Nutrients (2019) 11(12):2927. doi: 10.3390/nu11122927 PMC6950498 31816844

[28] Mikulska AA Filipowicz D Główka FK Szczepanek-Parulska E Ruchała M Bartecki M. HPLC analysis of the urinary iodine concentration in pregnant women. Molecules (2021) 26(22):6797. doi: 10.3390/molecules26226797 PMC8619590 34833891

[29] Andersen SL Sørensen LK Krejbjerg A Møller M Klitbo DM Nøhr SB. Iodine status in Danish pregnant and breastfeeding women including studies of some challenges in urinary iodine status evaluation. J Trace Elem Med Biol (2015) 31:285–9. doi: 10.1016/j.jtemb.2014.11.004 25535149

[30] Prevention CfDCa. national health and nutrition examination survey. Available at: https://wwwn.cdc.gov/Nchs/Nhanes/2005-2006/UIO_D.htm (Accessed 13th June 2022).

[31] Tayie FAK Jourdan K. Hypertension, dietary salt restriction, and iodine deficiency among adults. Am J Hypertens (2010) 23(10):1095–102. doi: 10.1038/ajh.2010.120 20559287

[32] Inoue K Leung AM Sugiyama T Tsujimoto T Makita N Nangaku M. Urinary iodine concentration and mortality among U. S Adults Thyroid (2018) 28(7):913–20. doi: 10.1089/thy.2018.0034 PMC6916127 29882490

[33] Francis EC Zhang L Witrick B Chen L. Health behaviors of American pregnant women: a cross-sectional analysis of NHANES 2007-2014. J Public Health (Oxford England) (2021) 43(1):131–8. doi: 10.1093/pubmed/fdz117 31832663

[34] Rattan P Penrice DD Ahn JC Ferrer A Patnaik M Shah VH. Inverse association of telomere length with liver disease and mortality in the US population. Hepatol Commun (2022) 6(2):399–410. doi: 10.1002/hep4.1803 34558851 PMC8793996

[35] Huang W Gong D Bao Y. Urinary iodine and serum 25-hydroxyvitamin d are associated with depression in adolescents. Trop J Pharm Res (2019) 17(12):2471–2476. doi: 10.4314/tjpr.v17i12.24

[36] Inker LA Schmid CH Tighiouart H Eckfeldt JH Feldman HI Greene T. Estimating glomerular filtration rate from serum creatinine and cystatin c. N Engl J Med (2012) 367(1):20–9. doi: 10.1056/NEJMoa1114248 PMC4398023 22762315

[37] Xie X Lu C Wu M Liang J Ying Y Liu K. Association between triclocarban and triclosan exposures and the risks of type 2 diabetes mellitus and impaired glucose tolerance in the national health and nutrition examination survey (NHANES 2013-2014). Environ Int (2020) 136:105445. doi: 10.1016/j.envint.2019.105445 31918332 PMC7027658

[38] National health and nutrition examination survey: 2007-2008 data documentation, codebook, and frequencies. Available at: https://wwwn.cdc.gov/Nchs/Nhanes/2007-2008/THYROD_E.htm#LBXT4F (Accessed March 17,2023).

[39] Ruan Z Lu T Chen Y Yuan M Yu H Liu R. Association between psoriasis and nonalcoholic fatty liver disease among outpatient US adults. JAMA Dermatol (2022) 158(7):745–53. doi: 10.1001/jamadermatol.2022.1609 PMC9134040 35612851

[40] Borel A-L Coumes S Reche F Ruckly S Pépin J-L Tamisier R. Waist, neck circumferences, waist-to-hip ratio: which is the best cardiometabolic risk marker in women with severe obesity? the SOON cohort. PloS One (2018) 13(11):e0206617. doi: 10.1371/journal.pone.0206617 30408116 PMC6224066

[41] Villatoro-Santos CR Ramirez-Zea M Villamor E. Urinary sodium, iodine, and volume in relation to metabolic syndrome in mesoamerican children and their parents. Nutr Metab Cardiovasc Dis (2022) 32(7):1774–83. doi: 10.1016/j.numecd.2022.04.022 35637087

[42] Jin M Zhang Z Li Y Teng D Shi X Ba J. U-Shaped associations between urinary iodine concentration and the prevalence of metabolic disorders: a cross-sectional study. Thyroid (2020) 30(7):1053–65. doi: 10.1089/thy.2019.0516 32188373

[43] Kim HJ Park S Park SJ Park HK Byun DW Suh K. Association between iodine intake and metabolic syndrome in euthyroid adult in an iodine-replete area: a nationwide population-based study. Endocr J (2022). doi: 10.1507/endocrj.EJ22-0389 36567075

[44] Herter-Aeberli I Cherkaoui M El Ansari N Rohner R Stinca S Chabaa L. Iodine supplementation decreases hypercholesterolemia in iodine-deficient, overweight women: a randomized controlled trial. J Nutr (2015) 145(9):2067–75. doi: 10.3945/jn.115.213439 26203098

[45] Xiao Y Sun H Li C Li Y Peng S Fan C. Effect of iodine nutrition on pregnancy outcomes in an iodine-sufficient area in China. Biol Trace Elem Res (2018) 182(2):231–7. doi: 10.1007/s12011-017-1101-4 28770411

[46] Larsson-Nyrén G Sehlin J. Anion-selective amplification of glucose-induced insulin secretion. Acta Diabetol (2002) 39(1):41–7. doi: 10.1007/s005920200011 12043938

[47] Menon VU Chellan G Sundaram KR Murthy S Kumar H Unnikrishnan AG. Iodine status and its correlations with age, blood pressure, and thyroid volume in south Indian women above 35 years of age (Amrita thyroid survey). Indian J Endocrinol Metab (2011) 15(4):309–15. doi: 10.4103/2230-8210.85584 PMC3193780 22029002

[48] García-Solís P Solís-S JC García-Gaytán AC Reyes-Mendoza VA Robles-Osorio L Villarreal-Ríos E. Iodine nutrition in elementary state schools of queretaro, Mexico: correlations between urinary iodine concentration with global nutrition status and social gap index. Arq Bras Endocrinol Metabol (2013) 57(6):473–82. doi: 10.1590/S0004-27302013000600010 24030188

[49] Farebrother J Dalrymple KV White SL Gill C Brockbank A Lazarus JH. Iodine status of pregnant women with obesity from inner city populations in the united kingdom. Eur J Clin Nutr (2021) 75(5):801–8. doi: 10.1038/s41430-020-00796-z 33184453

[50] Al-Attas OS Al-Daghri NM Alkharfy KM Alokail MS Al-Johani NJ Abd-Alrahman SH. Urinary iodine is associated with insulin resistance in subjects with diabetes mellitus type 2. Exp Clin Endocrinol Diabetes (2012) 120(10):618–22. doi: 10.1055/s-0032-1323816 23203253

[51] Lee KW Shin D Song WO. Low urinary iodine concentrations associated with dyslipidemia in US adults. Nutrients (2016) 8(3):171. doi: 10.3390/nu8030171 26999198 PMC4808899

[52] Liu M Li S-M Li X-W Wang P-H Liang P Li S-H. [Exploratory study on the association between high iodine intake and lipid]. Zhonghua Liu Xing Bing Xue Za Zhi (2009) 30(7):699–701. doi: 10.3760/cma.j.issn.0254-6450.2009.07.013 19957594

[53] Zhao S-J Ye Y Sun F-J Tian E-J Chen Z-P. The impact of dietary iodine intake on lipid metabolism in mice. Biol Trace Elem Res (2011) 142(3):581–8. doi: 10.1007/s12011-010-8767-1 20652651

[54] Banach W Nitschke K Krajewska N Mongiałło W Matuszak O Muszyński J. The association between excess body mass and disturbances in somatic mineral levels. Int J Mol Sci (2020) 21(19):7306. doi: 10.3390/ijms21197306 PMC7582962 33022938

[55] Serrano-Nascimento C Salgueiro RB Vitzel KF Pantaleão T Corrêa da Costa VM Nunes MT. Iodine excess exposure during pregnancy and lactation impairs maternal thyroid function in rats. Endocr Connect (2017) 6(7):510–21. doi: 10.1530/EC-17-0106 PMC5597975 28814477

[56] Leung AM Braverman LE. Consequences of excess iodine. Nat Rev Endocrinol (2014) 10(3):136–42. doi: 10.1038/nrendo.2013.251 PMC3976240 24342882

[57] Lee YK Kim JE Oh HJ Park KS Kim SK Park SW. Serum TSH level in healthy koreans and the association of TSH with serum lipid concentration and metabolic syndrome. Korean J Intern Med (2011) 26(4):432–9. doi: 10.3904/kjim.2011.26.4.432 PMC3245392 22205844

[58] Du F-M Kuang H-Y Duan B-H Liu D-N Yu X-Y. Associations between thyroid hormones within the euthyroid range and indices of obesity in obese Chinese women of reproductive age. Metab Syndr Relat Disord (2019) 17(8):416–22. doi: 10.1089/met.2019.0036 31355704

[59] Guo Y Hu C Xia B Zhou X Luo S Gan R. Iodine excess induces hepatic, renal and pancreatic injury in female mice as determined by attenuated total reflection Fourier-transform infrared spectrometry. J Appl Toxicol (2022) 42(4):600–16. doi: 10.1002/jat.4242 34585417

[60] Winkler R Griebenow S Wonisch W. Effect of iodide on total antioxidant status of human serum. Cell Biochem Funct (2000) 18(2):143–6. doi: 10.1002/(SICI)1099-0844(200006)18:2<143::AID-CBF857>3.0.CO;2-# 10814974

[61] Soriguer F Gutiérrez-Repiso C Rubio-Martin E Linares F Cardona I López-Ojeda J. Iodine intakes of 100-300 μg/d do not modify thyroid function and have modest anti-inflammatory effects. Br J Nutr (2011) 105(12):1783–90. doi: 10.1017/S0007114510005568 21262066

[62] Zbigniew S. Role of iodine in metabolism. Recent Pat Endocr Metab Immune Drug Discov (2017) 10(2):123–6. doi: 10.2174/1872214811666170119110618 28103777

[63] Chartoumpekis DV Ziros PG Georgakopoulos-Soares I Smith AAT Marques AC Ibberson M. The transcriptomic response of the murine thyroid gland to iodide overload and the role of the Nrf2 antioxidant system. Antioxidants (Basel) (2020) 9(9):884. doi: 10.3390/antiox9090884 PMC7555824 32961913

[64] National Institutes of Health. Iodine: fact sheet for health professionals. Available at: https://ods.od.nih.gov/factsheets/Iodine-HealthProfessional/ (Accessed March 15,2023).

