# Supplementary material for: Associations between urinary iodine concentration and the prevalence of metabolic disorders: a cross-sectional study
Source: Front Endocrinol (Lausanne). 2023 May 8;14:1153462. doi: 10.3389/fendo.2023.1153462 (PMC10200914; doi:10.3389/fendo.2023.1153462)
Supplement: Supplementary file 1 [file Table_1.docx]

**Table S1: Multifactor linear regression between BMI and UIC.**

| **Variable** | **Crude Model** | ***P* value** | **Model 1** | ***P* value** | **Model 2** | ***P* value** | **Model 3** | ***P* value** |
| --- | --- | --- | --- | --- | --- | --- | --- | --- |
|  | **coefficient (95%CI)** |  | **coefficient (95%CI)** |  | **coefficient (95%CI)** |  | **coefficient (95%CI)** |  |
| **UIC（log）** | 2.08 (1.698~2.462) | <0.001* | 2.06 (1.673~2.451) | <0.001* | 1.97(1.592~2.347) | <0.001* | 1.97(1.591~2.355) | <0.001* |
| **Classified UIC (mg/d)** | |  |  |  |  |  |  |  |
| **Normal UIC (n = 4064)** | 1(Ref) |  | 1(Ref) |  | 1(Ref) |  | 1(Ref) |  |
| **Low UIC (n = 5507)** | -1.35(-1.71~-0.99) | <0.001* | -1.34 (-1.699~-0.979) | <0.001* | -1.28 (-1.638~-0.930) | <0.001* | -1.29 (-1.644~-0.936) | <0.001* |
| **High UIC (n = 835)** | 0.56 (-0.10~1.22) | 0.096 | 0.53 (-0.136~1.200) | 0.117 | 0.61 (-0.059~1.286) | 0.073 | 0.63 (-0.052~1.309) | 0.070 |
| **Very high UIC (n = 1139)** | -0.07 (-0.58~0.44) | 0.781 | -0.09 (-0.605~0.420) | 0.721 | -0.15 (-0.635~0.338) | 0.546 | -0.14 (-0.625~0.341) | 0.560 |
| ***P* for trend** |  | 0.366 |  | 0.312 |  | <0.001* |  | 0.288 |

Data are expressed as weighted percentages. Coefficient and 95% CI for risk of metabolic syndrome and its components were estimated using complex samples linear regression.

* represents p < 0.05.

Crude Model: not adjusted;

Model 1: Adjusted for age, sex;

Model 2: Adjusted for age, sex, race/ethnicity, education, annual family income, smoking status, alcohol intake, physical activity,

Model 3: Adjusted for age, sex, race/ethnicity, education, annual family income, smoking status, alcohol intake, physical activity, thyroid problems, cancer, energy intake, fish or shellfish intake, sodium intake, eGFR, TSH, and FT4.

**Normal UIC: < 100ug/L**

**Low UIC: 100-299ug/L**

**High UIC: 300-399ug/L**

**Very high UIC: ≥ 400ug/L**
