# Supplementary material for: Associations between urinary iodine concentration and the prevalence of metabolic disorders: a cross-sectional study
Source: Front Endocrinol (Lausanne). 2023 May 8;14:1153462. doi: 10.3389/fendo.2023.1153462 (PMC10200914; doi:10.3389/fendo.2023.1153462)
Supplement: Supplementary file 2 [file Table_2.docx]

TableS2.Multiple logistic regression between UIC and MetS and its components. (participants with thyroid dysfunction were excluded)

| Variables | N (%) | crude model |  | Model 1 |  | Model 2 |  | Model 3 | *p* |
| --- | --- | --- | --- | --- | --- | --- | --- | --- | --- |
|  |  | OR (95%CI) | *p* | OR (95%CI) | *p* | OR (95%CI) | *p* | OR (95%CI) |  |
| **MetS** |  |  |  |  |  |  |  |  |  |
| **UIC (log10)** |  | 1.63(1.374,1.940) | <0.001* | 1.49(1.240,1.778) | <0.001* | 1.44(1.208,1.722) | <0.001* | 1.44(1.200,1.715) | <0.001* |
| **Classified UIC** |  |  |  |  |  |  |  |  |  |
| normal UIC | 1406(47.78) | ref |  | ref |  | ref |  | ref |  |
| low UIC | 861(32.75) | 0.80(0.687,0.939) | 0.006* | 0.85(0.725,0.992) | 0.040* | 0.86(0.734,1.004) | 0.056 | 0.86(0.734,1.005) | 0.059 |
| high UIC | 231(9.71) | 1.42(1.134,1.780) | 0.003* | 1.36(1.074,1.725) | 0.011* | 1.36(1.073,1.729) | 0.033* | 1.36(1.069,1.724) | 0.013* |
| very high UIC | 277(9.76) | 1.07(0.846,1.340) | 0.510 | 1.04(0.825,1.299) | 0.764 | 1.00(0.798,1.245) | 0.681 | 1.01(0.800,1.245) | 0.983 |
| *p for trend* |  |  | 0.292 |  | 0.437 |  | 0.531 |  | 0.557 |
| **MetS.Diabetes** |  |  |  |  |  |  |  |  |  |
| normal UIC | 1596(49.33) | ref |  | ref |  | ref |  | ref |  |
| low UIC | 1007(34.04) | 0.80(0.697,0.927) | 0.03* | 0.90(0.778,1.028) | 0.115 | 0.90(0.785,1.036) | 0.142 | 0.91(0.779,1.030) | 0.122 |
| high UIC | 247(8.93) | 1.21(0.938,1.565) | 0.139 | 1.10(0.832,1.466) | 0.490 | 1.12(0.845,1.483) | 0.428 | 1.12(0.850,1.492) | 0.416 |
| very high UIC | 247(7.70) | 0.75(0.583,0.960) | 0.023* | 0.67(0.519,0.857) | 0.002* | 0.66(0.514,0.851) | 0.002* | 0.65(0.506,0.843) | 0.001* |
| *p for trend* |  |  | 0.063 |  | 0.011* |  | 0.012* |  | 0.01* |
| **MetS.obesity** |  |  |  |  |  |  |  |  |  |
| normal UIC | 2836(47.32) | ref |  | ref |  | ref |  | ref |  |
| low UIC | 1941(35.52) | 0.86(0.753,0.970) | 0.016* | 0.85(0.750,0.967) | 0.014* | 0.86(0.757,0.974) | 0.019** | 0.86(0.755,0.974) | 0.018* |
| high UIC | 424(7.89) | 1.12(0.901,1.385) | 0.310 | 1.09(0.870,1.367) | 0.446 | 1.10(0.868,1.394) | 0.425 | 1.09(0.866,1.383) | 0.446 |
| very high UIC | 559(9.27) | 1.06(0.881,1.281) | 0.523 | 1.08(0.871,1.276) | 0.583 | 1.02(0.841,1.248) | 0.810 | 1.02(0.836,1.245) | 0.842 |
| *p for trend* |  |  | 0.714 |  | 0.838 |  | 0.980 |  | 0.977 |
| **MetS.hypertension** |  |  |  |  |  |  |  |  |  |
| normal UIC | 844(44.68) | ref |  | ref |  | ref |  | ref |  |
| low UIC | 648(38.98) | 1.09(0.925,1.290) | 0.293 | 1.22(1.030,1.440) | 0.022* | 1.22(1.027,1.444) | 0.024* | 1.22(1.033,1.450) | 0.020* |
| high UIC | 137(7.82) | 1.13(0.841,1.505) | 0.422 | 1.04(0.768,1.397) | 0.814 | 1.06(0.785,1.437) | 0.692 | 1.05(0.780,1.418) | 0.739 |
| very high UIC | 164(8.52) | 0.98(0.775,1.280) | 0.896 | 0.91(0.686,1.210) | 0.516 | 0.93(0.699,1.238) | 0.616 | 0.94(0.708,1.252) | 0.674 |
| *p for trend* |  |  | 0.681 |  | 0.996 |  | 0.849 |  | 0.808 |
| **MetS.low-HDL-C** |  |  |  |  |  |  |  |  |  |
| normal UIC | 1530(47.28) | ref |  | ref |  | ref |  | ref |  |
| low UIC | 1029(33.76) | 0.84(0.754,0.936) | 0.002* | 0.81(0.723,0.896) | <0.001* | 0.81(0.729,0.910) | <0.001* | 0.82(0.731,0.911) | <0.001* |
| high UIC | 255(8.88) | 1.28(1.015,1.623) | 0.037* | 1.33(1.042,1.695) | 0.023* | 1.31(1.038,1.682) | 0.024* | 1.33(1.038,1.693) | 0.025* |
| very high UIC | 314(10.08) | 1.15(0.961,1.377) | 0.124 | 1.18(0.987,1.418) | 0.069 | 1.14(0.950,1.374) | 0.156 | 1.13(0.941,1.366) | 0.184 |
| *p for trend* |  |  | 0.112 |  | 0.08 |  | 0.135 |  | 0.149 |
| **MetS.TG** |  |  |  |  |  |  |  |  |  |
| normal | 1852(47.15) | ref |  | ref |  | ref |  | ref |  |
| Low UIC | 1171(32.88) | 0.80(0.692,0.918) | 0.002* | 0.85(0.742,0.984) | 0.029* | 0.85(0.741,0.984) | 0.030* | 0.86(0.747,0.988) | 0.034* |
| high UIC | 314(9.13) | 1.40(1.133,1.731) | 0.002* | 1.33(1.066,1.658) | 0.012* | 1.32(1.061,1.654) | 0.014* | 1.32(1.0541.646) | 0.016* |
| very high UIC | 422(10.84) | 1.33(1.121,1.583) | 0.001* | 1.28(1.065,1.534) | 0.009* | 1.26(1.049,1.505) | 0.012* | 1.26(1.056,1.511) | 0.011* |
| *p for trend* |  |  | 0.006* |  | 0.016* |  | 0.022* |  | 0.019* |

UIC, urinary iodine concentration; MetS, metabolic syndromes; HDL, high-density lipoprotein; TG, triglyceride.

MetS.Diabetes, History of diabetes, Glycosylated hemoglobin (HbA1c) > 6.5%, fasting glucose (mmol/L) ≥ 7.0, or medication used to treat diabetes;

MetS.obesity, Waist Circumference (WC) ≥ 102 cm in men or ≥ 88 cm in women;

MetS.hypertension, blood pressure ≥ 130/85 mmHg or hypertension history or treatment with antihypertensive medication;

MetS.low-HDL-C, HDL-C < 40 mg/dL for men and < 50 mg/dL for women or use of anti-lipid abnormalities;

MetS.TG, TG ≥ 150 mg/dL, or pharmacological treatment of TG.

Normal UIC: < 100ug/L; Low UIC: 100-299ug/L; High UIC: 300-399ug/L; Very high UIC: ≥ 400ug/L.

Data are expressed as weighted percentages. OR and 95% CI for risk of metabolic syndrome and its components were estimated using complex samples logistic regression.

* represents p < 0.05.

Crude Model: not adjusted;

Model 1: Adjusted for age, sex;

Model 2: Adjusted for age, sex, race/ethnicity, education, annual family income, smoking status, alcohol intake, physical activity,

Model 3: Adjusted for age, sex, race/ethnicity, education, annual family income, smoking status, alcohol intake, physical activity, cancer, energy intake, fish or shellfish intake, sodium intake, eGFR, TSH, and FT4.
