# Supplementary material for: Associations between urinary iodine concentration and the prevalence of metabolic disorders: a cross-sectional study
Source: Front Endocrinol (Lausanne). 2023 May 8;14:1153462. doi: 10.3389/fendo.2023.1153462 (PMC10200914; doi:10.3389/fendo.2023.1153462)
Supplement: Supplementary file 3 [file Table_3.docx]

Table S3.Relationship between MetS and continuous variable UIC (log10) in different subgroups of participants.

| variable | Total [n (%)] | OR (95% CI) | *p* | *p* for interaction | |
| --- | --- | --- | --- | --- | --- |
| Gender |  |  |  | 0.844 |  |
| Female | 5740(49.72) | 1.505(1.232,1.839) | <0.001* |  |  |
| Male | 5805(50.28) | 1.575(1.261,1.968) | <0.001* |  |  |
| Ethnic |  |  |  | 0.908 |  |
| Non-Hispanic White | 5138(44.5) | 1.549(1.125,2.134) | 0.008* |  |  |
| Mexican American | 1813(15.7) | 1.506(1.223,1.855) | <0.001* |  |  |
| Non-Hispanic Black | 2368(20.51) | 1.691(1.116,2.003) | 0.003* |  |  |
| Other Race | 2226(19.28) | 1.480(1.106,1.980) | 0.009* |  |  |
| Thyroid disease |  |  |  | 0.516 |  |
| yes | 1216(10.53) | 1.764(1.159,2.683) | 0.009* |  |  |
| no | 10329(89.47) | 1.497(1.264,1.772) | <0.001* |  |  |
| Age |  |  |  | 0.003* |  |
| ≥60 | 3925(34) | 1.163(0.905,1.493) | 0.234 |  |  |
| <60 | 7620(66) | 1.739(1.456,2.076) | <0.001* |  |  |

Data are expressed as weighted percentages. OR and 95% CI for risk of metabolic syndrome and its components were estimated using complex samples logistic regression.

* represents p < 0.05.

UIC, urinary iodine concentration; MetS, metabolic syndromes.

Adjusted for education, annual family income, smoking status, alcohol intake, physical activity, cancer, energy intake, fish or shellfish intake, sodium intake, eGFR, TSH, and FT4.
